# Expression of *AMELX*, *AMBN*, *ENAM*, *TUFT1*, *FAM83H* and *MMP20* Genes in Buccal Epithelial Cells from Patients with Molar Incisor Hypomineralization (MIH)—A Pilot Study

**DOI:** 10.3390/ijms26020766

**Published:** 2025-01-17

**Authors:** Wojciech Tynior, Dorota Hudy, Karolina Gołąbek, Agnieszka Raczkowska-Siostrzonek, Joanna Katarzyna Strzelczyk

**Affiliations:** 1 Department of Medical and Molecular Biology, Faculty of Medical Sciences in Zabrze, Medical University of Silesia in Katowice, 19 Jordana St., 41-808 Zabrze, Poland; 2 Department of Dental Surgery, Faculty of Medical Sciences in Zabrze, Medical University of Silesia in Katowice, 17 Plac Akademicki, 41-902 Bytom, Poland

**Keywords:** MIH, molar incisor hypomineralization, genes expression, *AMELX*, *AMBN*, *ENAM*, *TUFT1*, *FAM83H*, *MMP20*

## Abstract

Molar incisor hypomineralization (MIH) is a developmental defect that affects the enamel tissue of permanent teeth. Clinicians may observe a range of opacities in the affected teeth, varying from white to creamy, yellow, and brown. Of particular interest is an etiology of MIH that has not been rigorously elucidated. Researchers believe that there are many potential etiological factors with strong genetic and/or epigenetic influence. The primary factors contributing to the risk of MIH development include specific medical conditions and circumstances. These encompass prematurity, cesarean delivery, perinatal hypoxia, and various health issues such as measles, urinary tract infections, otitis media, gastrointestinal disorders, bronchitis, kidney diseases, pneumonia, and asthma. Although genetic research in this area has received substantial attention, the investigation of epigenetic factors remains comparatively underexplored. Special attention is given to genes and their protein products involved in amelogenesis. Examples of such genes are *AMELX*, *AMBN*, *ENAM*, *TUFT1*, *FAM83H*, and *MMP20*. The median relative *FAM83H* gene expression in the control group was 0.038 (0.031–0.061) and 0.045 (0.032–0.087) in the study group in buccal swabs. The median relative *TUFT1* gene expression in the control group was 0.328 (0.247–0.456) and 0.704 (0.334–1.183) in the study group in buccal swabs. Furthermore, children with MIH had significantly higher *TUFT1* expression levels compared to the control group (*p*-value = 0.0043). Alterations in the expression of the *TUFT1* and *FAM83H* genes could be contributing factors to MIH pathogenesis. Nonetheless, further investigation is essential to comprehensively elucidate the roles of all analyzed genes in the pathogenesis of MIH.

## 1. Introduction

Molar incisor hypomineralization (MIH) is a developmental defect affecting the enamel tissue of permanent teeth. The prevalence of this condition varies worldwide, ranging from 12.9% to 14.2% [1,2]. However, these data could be an underestimation. MIH is diagnosed if at least one permanent first molar is affected. The condition’s severity can differ significantly. Intraoral examination reveals a wide range of opacities in the affected teeth, varying from white to creamy, yellow, and brown, and demarcated opacities and post-eruptive enamel breakdown can be seen [3]. The clinical picture of severe MIH is often accompanied by hypersensitivity of the teeth while brushing and consuming hot/cold meals and beverages. Early detection and diagnosis of MIH is crucial. It can have a significant impact on children’s oral health due to the rapid progression of caries in MIH-affected teeth. The researchers are notably interested in understanding the etiology of MIH, which has not been fully clarified. It is hypothesized that multiple factors may contribute to the process of amelogenesis, with a particular emphasis on genetic and epigenetic influences [3,4,5]. Current research primarily focuses on environmental factors (respiratory issues, malnutrition, food intolerance, infection and medication intake) and genes directly involved in amelogenesis [4,5], while there is a growing interest in investigating genes linked to immune response pathways [6].

Under physiological conditions, the process of amelogenesis is a part of tooth development that starts prenatally. Enamel formation requires many ectomesenchymal interactions [7]. The inner enamel epithelium differentiates into ameloblasts secreting enamel matrix proteins. The next stage is the calcification of the protein structure and the organization of the enamel into units—namely, prisms [8]. This process is coordinated by many genes and their protein products. This knowledge was obtained from observations of developmental defects of enamel (DDEs) that cause hypoplasia or hypomineralization of the enamel. Phenotypic abnormalities in the teeth allow us to detect changes in the genotype, most commonly gene mutations and single nucleotide polymorphisms (SNPs). There are reports that mutations and SNPs of *AMELX* (Amelogenin X-Linked), *AMBN* (Ameloblastin), *ENAM* (Enamelin), *TUFT1* (Tuftelin 1), *FAM83H* (Family With Sequence Similarity 83 Member H), and *MMP20* (Matrix Metallopeptidase 20) genes, cause different nonsyndromic enamel conditions such as amelogenesis imperfecta (AI) [9,10,11,12].

Amelogenin is a protein that is essential for enamel formation and is produced by *AMELX* and *AMELY* genes. This protein is highly conserved across different species. The genes that code amelogenin are located on both the X and Y chromosomes, but 90% of the activity comes from *AMELX*, which is located on the X chromosome. The remaining 10% of amelogenins are produced by *AMELY*, which is located on the Y chromosome [13]. Amelogenins are expressed in preameloblasts and ameloblasts, and they play a vital role in coordinating the elongation and growth of hydroxyapatite prisms, the hard component of the enamel [14]. Mutations in the *AMELX* gene can result in hypoplasia or hypomineralization of the enamel. Some studies have confirmed that *AMELX* gene mutation is linked to one DDE—AI [15,16].

Ameloblastin is the second enamel matrix protein (EMP), apart from the *AMELX* gene, crucial for amelogenesis. It is involved in the mineralization and structural organization of enamel. The *AMBN* gene is located on chromosome 4 and encodes a 447 amino acid protein rich in proline. *AMBN* gene mutations lead to developmental abnormalities in the enamel [15,17].

Enamelin is the largest protein, with 1142 amino acids, that belongs to the EMP family. The *ENAM* gene is located on chromosome 4. The function of the protein is to form tooth enamel from developing crystals, but the exact mechanism is not fully explained. The mutations in the *ENAM* gene manifest as autosomal-dominant AI [16].

Tuftelin 1 was the first protein detected from the extracellular enamel by Deutsch et al. [18]. The *TUTF1* gene is located on chromosome 1. TUFT1 protein occurred near dentino-enamel junction (DEJ). The exact mechanism in amelogenesis is not known, but the function of the protein is to form enamel from developing crystals. *TUFT1* expression was found not only in mineralized tissue but also in non-mineralized tissue and cancer samples [12]. TUFT1 is involved in several biological signaling pathways, including hypoxia, mesenchymal stem cell function, neurotrophin nerve growth factor-mediated neuronal differentiation, metastasis, and tumor growth [19].

*FAM83H* is located on chromosome 8. Mutations of this gene can cause autosomal-dominant hypocalcified amelogenesis imperfecta (ADHCAI). Its activity is crucial for the proper formation of enamel [11]. However, its activity is also presented in different tumors: lung adenocarcinoma, lung large-cell carcinoma, lung squamous cell carcinoma, breast invasive ductal carcinoma, breast ductal carcinoma in situ, colon adenoma, hepatocellular carcinoma, as well as ovarian, pancreatic, and stomach cancers [20,21].

Matrix Metalloproteinase 20 gradually degrades the enamel proteins responsible for separating the thin crystal ribbons of the secretory stage. As a result of this process, the space left by the lost protein promotes crystal development. Mutations in the *MMP20* gene can implicate AI [22].

Studies concerning DDEs and their development in the prenatal period have been performed mainly on animals (mice and rats) and cellular models [23,24,25,26,27,28,29,30]. The main limitation of research on human tissues is the ethical considerations related to the treatment strategy and the inability to obtain DDE-affected teeth [3]. It is important to note that permanent molars are not typically extracted before orthodontic treatment. Therefore, the study of expression in another type of tissue—epithelial tissue—was considered to be appropriate.

The aim of the study is to explore the etiology of MIH by selected gene expression analysis in buccal epithelial cells of patients with MIH compared to healthy controls and to examine the possible association and correlation between the expression levels of genes with potential etiological factors.

## 2. Results

### 2.1. Gene Expression in Buccal Epithelial Cells

**Figure 1:**
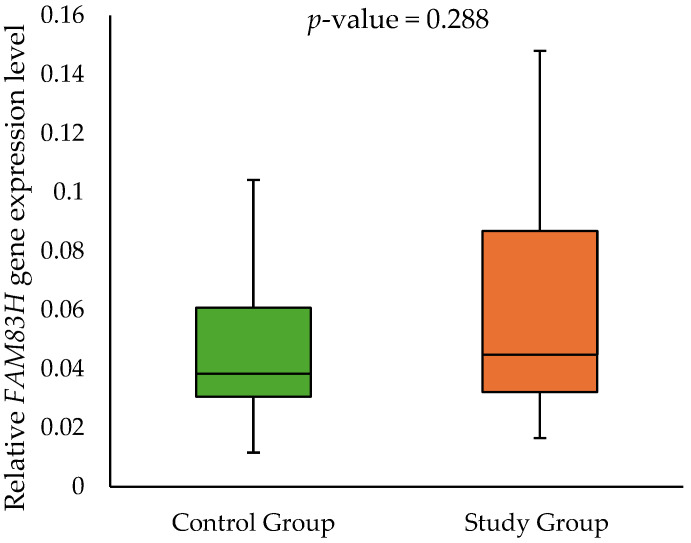
The relative *FAM83H* gene expression in the buccal epithelial specimens in the control group and study group.

**Figure 2:**
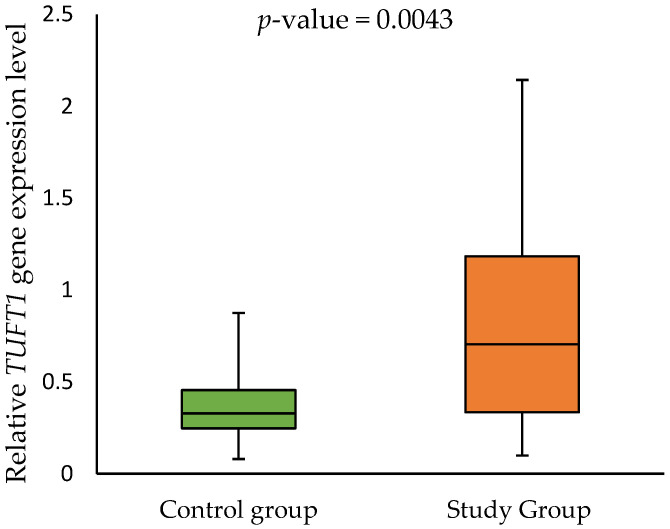
The relative *TUFT1* gene expression in the control group and study group.

The expression analysis of the genes *AMELX*, *AMBN*, *ENAM*, *TUFT1*, *FAM83H*, and *MMP20*, separated into two groups (children diagnosed with MIH being the study group and children without MIH as the control group), showed the expression of only two of them—*FAM83H* and *TUFT1*. The median relative *FAM83H* gene expression in the control group was 0.038 (0.031–0.061) and 0.045 (0.032–0.087) in the study group. Meanwhile, the median relative *TUFT1* gene expression in the control group was 0.328 (0.247–0.456) and 0.705 (0.334–1.183) in the study group. Furthermore, children with MIH had significantly higher *TUFT1* expression levels in comparison to the control group (*p*-value = 0.0043). These results are shown in Figure 1 and Figure 2.

We also assessed the correlation of the expression levels of selected genes with possible etiological MIH factors such as age of the patients, age of the parents, time of the delivery, birth weight, and birth body length. There were no significant associations between the relative gene expression and variables of possible etiological factors, except for the age of patients. We observed in the study group that the *FAM83H* expression levels were correlated positively (R^2^ = 0.575, *p*-value = 0.003) with the age of patients. Results are presented in Table 1.

### 2.2. Additional Analyses of Gene Expressions in Tooth Tissue Samples

The expression analysis of the genes *AMELX*, *AMBN*, *ENAM*, *TUFT1*, *FAM83H*, and *MMP20* in all teeth showed the expression of only four of them, i.e., *AMBN*, *TUFT1*, *FAM83H*, and *MMP20*. We found no statistically significant differences in gene expressions in teeth without caries compared to teeth with caries. Results are presented in Table 2.

Gene expressions in a single MIH-affected tooth are shown in Table 3.

We observed in the entire control group that the *MMP20* gene’s expression levels were negatively correlated (R^2^ = −0.699, *p*-value = 0.017) with patient’s age.

## 3. Discussion

To our knowledge, the present study is the first to investigate the expression of the analyzed genes (*AMELX*, *AMBN*, *ENAM*, *TUFT1*, *FAM83H*, and *MMP20*) in buccal epithelial cells from patients with MIH.

The most interesting observation emerging from our data was that children with MIH had significantly higher the *TUFT1* gene expression levels in comparison to the control group. In our study, the *TUFT1* gene expression level from buccal swabs of MIH-affected children was 2.1 times higher than in the control group. Due to the pioneering nature of the study, it is difficult to compare the gene expressions of our patients with MIH to other studies concerning MIH-affected children. Interestingly, increased *TUFT1* gene expression was observed in an animal model during hypoxia in kidneys, testes, and brain tissues [31,32]. Adaptation of tissues and cells to hypoxic conditions results in the activation of genes, including those responsible for angiogenesis, iron metabolism, glucose metabolism, and cell proliferation/survival. The main mediator of this process is hypoxia-inducible factor-1α (HIF-1α). The dysfunction of HIF-1α has been associated with abnormalities in dentinogenesis and amelogenesis [33]. Sidaly et al. found that the expression of structural genes on mice cell lines. The expressions of *AMELX*, *AMBN*, *ENAM*, and *MMP20* genes were increased after 24 and 48 h of hypoxia [34]. However, the *TUFT1* gene was not tested in that research. Moreover, children with hypoxia-related factors showed a higher incidence of hypomineralized second primary molars—a similar condition to MIH, developed in primary dentition. The condition of hypoxia during delivery is also an influential risk factor for the development of MIH in children (OR 2.76; 95% CI 2.09–3.64; *p* < 0.0001) [35]. It could be presumed that hypoxia during the delivery of MIH-affected children might be an influential factor in the different expression levels of *TUFT1*. However, such a hypothesis requires targeted studies in a larger group of patients and remains a hypothesis in the current state of knowledge.

The *FAM83H* gene is expressed during amelogenesis and in other tissues including the eyes, kidneys, liver, bladder, larynx, and cervix [36,37]. Different mutations of the *FAM83H* gene are involved in AI—a similar condition to MIH in a group of DDEs. This gene overexpression has a strong impact on ADHCAI development [36,38,39,40]. The study by Kuga and others has shown an interesting observation. The *FAM83H* gene is involved in regulating keratin cytoskeleton organization. Overexpression of *FAM83H* disassembles keratin filaments. Clinically observed aberrations occur in colorectal cancer, myeloma, and AI [41,42,43]. Concerning AI, among affected individuals, the protein was localized in the nuclei of cells, as opposed to healthy individuals in whom the protein was in the cytoplasm [44,45]. Moreover, a knockout of the *FAM83H* gene caused a significant reduction in the expression levels of *AMBN*, *MMP20*, *DSPP*, and *FGF10* genes in the dental root in a mouse model [46]. In our study, the expression of the *FAM83H* gene in buccal, although not statistically different, was presented in the group diagnosed with MIH (the study group) and children without MIH. We observed a positive correlation of the *FAM83H* gene with the age of patients in epithelial samples. *FAM83H* gene expression was also observed in tooth samples. However, this result refers to adults without MIH—i.e., the control group.

The *AMBN* gene is involved in amelogenesis as one of the key genes responsible for controlling enamel thickness. It controls the mineral phase, involving the transformation, elongation, and organization of calcium hydroxyapatite crystals into rods [47]. Some mutations of the *AMBN* gene are well-documented factors in AI and also in epithelial odontogenic tumors [15,28,38,39,40,48]. Expression analysis of *AMBN* in AI has not been performed. Chun et al. performed gene expression analyses in an animal model using transgenic mice with different genotypes of the *AMBN* gene (wild-type and models representing *AMBN* underexpression and overexpression) [49]. The mice were housed under unrestricted access to food and water for 7 weeks. The animals with *AMBN* overexpression presented MIH-like symptoms on their teeth: demarcated defects, opaque color, and reduced mineral content spanning the width of the enamel, starting at the innermost enamel and exhibiting enamel breakdown at the DEJ. In our study, buccal samples did not reveal *AMBN* expression. *AMBN* gene expression was observed only in tooth specimens. The only result we registered was that the expression of *AMBN* negatively correlated with the age of two control groups of patients with tooth specimens. However, this result relates to adults without MIH.

The MMP20 protein is crucial for proper tooth development [22]. As a proteolytic enzyme, it is mainly involved in the secretory phase of amelogenesis [50,51]. It is active during the secretory phase of amelogenesis when it cleaves amelogenin. It generates the space for hard mineral structure and organizes the growth of crystals [22]. MMP20 expression is present in the ostellate reticulum, the stratum intermedium, the secretory ameloblasts, the odontoblasts, and the dental papilla [52]. A disruption in the activity of this gene leads to the development of autosomal recessive hypomaturation AI [53]. Enamel deformation associated with changes in *MMP20* expression was also confirmed in a mouse model [54]. In our study, the expression of the *MMP20* gene was revealed among two control groups, and for adolescents, only in dental samples. We also observed that the expression of *MMP20* negatively correlated with the age of patients in dental tissues.

*AMELX* is expressed in various tissues such as the eyes, ovary, dental epithelial cells, and dental mesenchymal cells in mouse models. Jacquea et al., using the RT-PCR method, showed that *AMELX* expression was low in the alveolar and basal bones. Furthermore, *AMELX* mRNA levels in tissues varied depending on two parameters: first, the stage of ontogenesis, decreasing with age, and second, tissue type (e.g., a higher level in dental epithelial cells and alveolar bone when compared to basal bone and dental mesenchymal cells in 1-week-old mice) [55]. The expression of the *AMELX* gene was not presented in our study.

*ENAM* gene mutations are highly involved in the development of AI. Different mutations are responsible for some forms of AI, namely autosomal dominant and autosomal recessive [56]. An example of a mutation and therefore a change in gene expression is the splicing donor site mutation (NM_031889.3: c.-61 + 1G > A) [57]. There is also a different mechanism that regulates *ENAM* gene expression. The reduction in *ENAM* gene expression was observed in *Msx2*-deficient mice. Incisors and molars in their pre-secretory and secretory stages of amelogenesis require the *MSX2* gene for proper *ENAM* gene expression [58]. In our study, no *ENAM* gene expression was detected in either the epithelium or the teeth.

A limitation of the present study is the number of epithelial samples and the number of tooth specimens included in the additional analyses. Further studies on larger and more diverse groups are warranted to better understand the significance of the expression of selected genes and their role in MIH.

## 4. Materials and Method

### 4.1. Ethical Considerations

This study was conducted in accordance with the ethical standards of the Medical University of Silesia in Katowice and adhered to the principles outlined in the Declaration of Helsinki. Ethical approval was obtained (PCN/0022/KB1/108/I/19/20 and PCN/0022/KB1/108/II/19/20) on 5 May 2020. Participants were fully informed about the study’s purpose, procedures, potential risks, and benefits. Written informed consent was obtained from all participants/parents/legal guardians before their inclusion in the study. To ensure confidentiality, all personal identifiers were removed from the digital data set. Data were stored securely on password-protected servers, and access was limited to the research team. Participation in the study was voluntary. Participants were informed of their right to withdraw at any stage without any consequences.

### 4.2. Clinical Examination

The presence of MIH was based on the judgment criteria presented by The European Academy of Paediatric Dentistry (EAPD) [59]. The dental practitioners were familiar with the EAPD guidelines and trained to avoid misdiagnosis.

Before the swab collection procedure, an interview and dental examination was conducted to evaluate the morphology of the teeth. The children who met the inclusion criteria were classified into two groups: the study group—children diagnosed with MIH—and the control group—children without MIH. Children with dental enamel defects, cranio-mandibular disorders, and genetic disorders were excluded from the study.

### 4.3. Sample Collection

The tissue sampling protocol involved the collection of buccal mucosal epithelium specimens using a sterilized cotton swabs (Deltalab, Barcelona, Spain). Dentists used sterile swabs to scrape the exfoliated oral mucosa cells from the right and left cheeks of the patients. The collected material was protected from degradation by storage at sub-zero temperatures for further laboratory testing in fixRNA (Eurx, Gdańsk, Poland).

Teeth were collected from routine extraction from a child with MIH and adult patients. The study group consisted of one MIH-affected tooth extracted due to the destruction and hypersensitivity of the crown. The first control group consisted of first permanent molars obtained from adults. Inclusion criteria were deep caries with pulp chamber involvement and periapical infection. The second control group consisted of impacted third permanent molars extracted for orthodontic indications or recurrent inflammation. Due to the small study group—a single tooth with MIH—no statistical analyses were conducted, and only descriptive statistics of the groups were presented. Table 4 and Table 5 present the characteristics of the groups.

### 4.4. RNA Isolation from Sterile Swabs

RNA from swabs was isolated with Universal RNA Purification Kit (Eurx, Gdańsk, Poland) according to the manufacturer’s instructions. After isolation, RNA was stored at −80 °C until further analysis.

### 4.5. RNA Isolation from Teeth

Before RNA isolation, teeth were pulverized with mortar and pestle in liquid nitrogen and suspended in RNA Extracol (Eurx, Gdańsk, Poland). RNA was isolated with Universal RNA Purification Kit (Eurx, Gdańsk, Poland) according to the manufacturer’s instructions with RNA Extracol (Eurx, Gdańsk, Poland). After isolation, RNA was stored at −80 °C until further analysis.

### 4.6. Complementary DNA (cDNA) Synthesis

Total RNA (5 ng) was transcribed into cDNA using High Capacity cDNA Reverse Transcription Kit with RNase Inhibitor (Applied Biosystems, Foster City, CA, USA) on Mastercycler personal (Eppendorf, Hamburg, Germany). The reaction was performed in 20 μL volume containing 2 μL of 10× Buffer RT; 0.8 μL of 25× dNTP mix (100 mM); 2 μL of 10× RT Random Primers; 1 μL MultiScribe™ Reverse Transcriptase; 1 μL RNase inhibitor; 3.2 μL nuclease free H_2_O; and 10 μL RNA. The reaction was carried out in Mastercycler personal (Eppendorf, Hamburg, Germany) with the following thermal profile: 25 °C for 10 min, 37 °C for 120 min, 85 °C for 5 min, and 4 °C–∞.

### 4.7. Gene Expression Analysis

The relative genes expression (RQ) analysis was performed by Real-Time PCR (qPCR) using TaqMan^TM^ Gene Expression Assays, QuantStudio 5 RealTime PCR System and Analysis Software v1.5.1 (Applied Biosystems, Foster City, CA, USA). The qPCR was performed in a volume of 20 µL using 1 µL of cDNA, 10 µL of TaqMan^TM^ Fast Advanced Master Mix (Applied Biosystems, Foster City, CA, USA), 1 µL of TaqMan^TM^ Gene Expression Assays (Assay ID: Hs00365791_m1 for *AMELX*, Assay ID: Hs00212970_m1 for *AMBN*, Assay ID: Hs00929008_m1 for *ENAM*, Assay ID: Hs00360629_m1 for *TUFT1*, Assay ID: Hs00294647_s1 for *FAM83H*, Assay ID: Hs01573770_m1 for *MMP20*, and Assay ID: Hs03929097_g1 for *GAPH*), and 8 µL of nuclease free H_2_O (EURx, Gdańsk, Poland). The glyceraldehyde-3-phosphate dehydrogenase gene (*GAPDH*) was used as a reference gene. The reference gene was selected based on the literature [30,60]. The relative expression of genes was calculated from 2^−ΔΔCT^ according to Livak et al. [61]. The thermal cycle for all analyzed genes was 95 °C for 20 s, followed by 40 cycles of 95 °C for 1 s and 60 °C for 20 s.

### 4.8. Statistical Analysis

Assessment of data distribution was performed with the Shapiro–Wilk test. Results are presented as median with range, and the U Mann–Whitney test was used to determine the significance of differences between groups. Correlation of expression data with etiological factors was conducted with the Spearman coefficient rank. Differences or correlations with a *p*-value < 0.05 were regarded as significant. Analysis was performed using STATISTICA 13.1 (TIBCO Software Inc., Palo Alto, CA, USA).

## 5. Conclusions

Our pilot study is the first to evaluate selected gene expression in a group of children with MIH and control group. Alterations in the expression of the *TUFT1* and *FAM83H* genes could be contributing factors to MIH pathogenesis. The involvement of other genes in the pathogenesis of MIH cannot also be ruled out, taking into account the tissue-specific nature of gene expression. Nonetheless, further investigation is essential to comprehensively elucidate the roles of all analyzed genes in the pathogenesis of MIH.

## Figures and Tables

**Table 1:** The correlation between potential etiological factors for MIH and the expression levels of genes *TUFT1* and *FAM83H*.

Possible Etiological MIH Factors	Correlation Coefficient	
	*TUFT1*	*FAM83H*
Age of patient	0.002	0.575
Mother’s age	−0.117	−0.118
Father’s age	0.034	0.034
Time of delivery	0.003	0.036
Birth weight	0.116	−0.108
Birth body length	0.063	−0.103

**Table 2:** The relative *AMBN*, *TUFT1*, *FAM83H*, and *MMP20* gene expressions in teeth with caries and without caries.

The Relative Gene Expression Me (Q1–Q3)			
Gene	Control Group (MIH-) Permanent Molars Without Caries	Control Group (MIH-) Permanent Molars with Caries	*p*-Value
*AMBN*	0.0226 (0.0056–0.0325)	0.0016 (0.0006–0.0052)	0.095
*TUFT1*	0.0044 (0.0018–0.0055)	0.0030 (0.0017–0.0046)	0.788
*FAM83H*	0.0047 (0.0034–0.0066)	0.0088 (0.0047–0.0365)	0.202
*MMP20*	0.0373 (0.0111–0.0616)	0.0045 (0.0004–0.0163)	0.052

Me, median; Q1, lower quartile; Q3, upper quartile.

**Table 3:** The relative *AMBN*, *TUFT1*, *FAM83H*, and *MMP20* gene expression in a single MIH-affected tooth.

Gene	The Relative Gene Expression
*AMBN*	0.0223
*TUFT1*	0.0030
*FAM83H*	0.0063
*MMP20*	0.0687

**Table 4:** Characteristics of the groups collecting buccal specimens.

Buccal Specimens	Study Group (MIH+)	Control Group (MIH-)	*p*-Value
Number of samples	27	31	
Age (mean)	8.33	8.94	>0.05
Female/Male	11/16	14/17	>0.05

**Table 5:** Characteristics of the groups collecting tooth tissue.

Tooth Tissue	Study Group (MIH+)	Control Group (MIH-) Permanent Molars with Caries	Control Group (MIH-) Permanent Molars Without Caries
Number of tooth samples	1	5	7
First permanent molars/third permanent molars	1/0	2/3	0/7
Age (mean)	8	31.8	24.43
Female/male	1/0	4/2	7/0

## Data Availability

The collected data files are available on request from the corresponding author.
